# Key principles to support occupational engagement in mental health inpatient units

**DOI:** 10.1177/15691861251328585

**Published:** 2025-04-23

**Authors:** Jessica Levick, Kieran Broome, Florin Oprescu, Marion Gray

**Affiliations:** 1 95789 University of Southern Queensland, Australia; 2 University of Southern Queensland, Australia; 3 598088 Good to Better, Australia; 4 University of the Sunshine Coast, Australia

**Keywords:** Leisure, occupational therapy, mental health

## Abstract

**Introduction:**

Leisure activity is known to be health promoting and health creating. In mental health inpatient settings, leisure is a common activity that consumers can participate in regardless of their acuity. Leisure can be a powerful therapeutic modality harnessed by the entire multidisciplinary team.

**Methods:**

The Levick’s Leisure Practice Principles were developed based on the findings of Levick et al., (2022) and piloted with inpatient health staff. This included of seven nurses, one occupational therapist, two psychologists, and two psychiatrists (registrar and consultant).

**Results:**

Key evidence informed principles were developed to support occupational engagement in mental health inpatient units in Australia. Ten principles were created to support organisations to improve the quality of their inpatient settings by improving occupational opportunity.

**Findings:**

Currently no leisure principles exist for consumers to participate in mental health settings. The principles incorporate public health theory of salutogenesis and occupational therapy theory to generate evidence informed practice to promote recovery.

## Introduction

Leisure activity is known to be salutogenic (health-creating) and contributes to individuals’ quality of life (Chen & Chippendale, 2018). For the past 30 years, leisure has been identified in the literature as important and health-promoting in mental health settings (Chen & Chippendale, 2018). It is acknowledged the link between leisure and mental health is an emerging field, which is no less than any other areas in mental health. The World Health Organization (2021) suggests leisure activity and recreation are integral to inpatient care. Similarly, the National Mental Health Standards (Australia) (Australian Government, 2010) indicate leisure and recreational opportunities should be available for all consumers.

In occupational therapy, leisure is a powerful therapeutic modality with efficacy in mental health inpatient settings (Cutler et al., 2021; Lloyd & Lee Williams, 2010; Marshall et al., 2020). The core scope of practice of an occupational therapist is to utilise meaningful occupation to improve occupational performance and participation. The key to leisure being used effectively as a treatment modality is targeting activity that is meaningful to the individual, which focuses on the individual interests, volition, and values, drawing from the Model of Human Occupation (MOHO) (Taylor, 2017). Engagement in meaningful activity can assist in reducing incidences of aggression, seclusion, and restraint (Todman, 2003).

The issue of a lack of leisure activity in mental health inpatient units (MHIUs) MHIUs has been discussed in the literature for more than 20 years and there are limited clinical guidelines or references to the application in clinical settings (Foye et al., 2020; Morrison et al., 1996). A focus in MHIUs is to reduce seclusion and restraint with ‘least restrictive practices’, but a key factor to consumers reporting boredom is they remain unoccupied with the dearth of activity offered (Marshall et al., 2020; Wilson et al., 2018).

Levick et al. (2022) established that staff and consumers would benefit from more occupational opportunities, social connections, and a sense of community in locked settings. With consideration of the MOHO, the built and social environment are key barriers to engagement in meaningful occupation in a mental health setting. Currently, there is limited evidence-informed principles that can guide service delivery to offer meaningful occupation and intervention that facilitate participation and recovery.

A list of 10 practice principles called Levick’s Leisure Practice Principles were created in response to a theoretical gap indicating limited occupation-focused guidelines for inpatient mental health settings. There are several clinical guidelines set around the world exploring optimal care of mental health consumers, but limited exploring patient-centred therapeutic engagement that promotes recovery. The practice principles provided a number of valid arguments that leisure is salutogenic and beneficial for people’s mental health. A lack of leisure activity continues to be an ongoing problem, fostering boredom and in turn creating occupational deprivation for those with prolonged admissions. There is an increase in the emphasis and importance of leisure time in acute mental health settings, which is considered health creating and health-promoting. Therefore, MHIUs should consider broadening their leisure programs to daily activity and expanding resources available as the bare minimum to improve the consumer experience.

## Development

The Levick’s Leisure Practice Principles were developed through PhD research exploring the therapeutic benefits of leisure in mental health inpatient units (Levick et al., 2022). This body of research (Levick et al., 2022) on Australian mental health inpatient units indicated the need for a focus on the development of practice principles, which is a list of key values and learnings to support the implementation of leisure activity in mental health inpatient units. This research adopted similar practices from Brown et al. (2013) who developed a list of evidence-based recommendations for adult physical therapy patients and Brownie (2011) who created the Eden Principles for aged care. A list of practice principles has been developed based on the findings of Levick et al. (2022) to guide clinicians and governing bodies on how to improve service delivery of leisure. Levick et al. (2022) conducted a literature review and a series of mixed methods descriptive studies. This included content analysis of staff’s perspectives of consumer engagement in leisure inpatient units; a mixed method survey (including three standardised tools MHSIP, CLIP, and the Leisure Boredom Scale) of consumers perspectives of what should be included for occupational opportunity in inpatient settings; a policy analysis of Australian mental health act legislation; and the development of a leisure tool called the Checklist of Leisure, Interest and Participation (CLIP) to assess leisure interests.

The concept of occupational enrichment and occupational deprivation were key theoretical foundations and considerations during development (Whiteford et al., 2020). Occupational enrichment is considered the goal for optimal function if a person is experiencing occupational deprivation (Whiteford et al., 2020). The practice principles will be based on the concept of occupational enrichment and be supported by the feedback from consumers (Levick et al., 2023), stakeholders, and policy/legislation (Levick et al., 2022). The concept of occupational enrichment (Whiteford et al., 2020) is the intention and goal of the principles.

A review of the Australian National Mental Health Standards Australian Government (2010) and recovery model principles (Commonwealth of Australia, 2013) ensured the practice principles complemented mental health models/frameworks currently in place across acute settings which are applicable to an Australian Mental Health Context. The development of the practice principles aims to generate discussion amongst policymakers and change in governing policy and legislation.

The goal of compiling the practice principles was to generate recommendations that governing bodies could implement in MHIUs to create immediate change. Some key areas that MHIUs may focus on are the built environment, social connection (Wilcock, 1995), policy and legislation, and promotion of occupational opportunity (Molineux & Whiteford, 1999). The practice principles will support and promote consumers to engage in their environment and aim to understand their perspective (Brown et al., 2013; Liamputtong, 2017).

## Pilot

The Levick’s Leisure Practice Principles were shared with a group of stakeholders to ascertain feedback on whether the recommendations were practical, realistic, and meaningful to the context. The stakeholder group was formed to gain industry feedback on the utility in a clinical setting and receptiveness of implementing leisure evidence-based practice principles. The mental health clinicians who provided feedback consisted of seven nurses, one occupational therapist, two psychologists, and two psychiatrists (registrar and consultant). All stakeholders identified the practice principles as “crucial”, “necessary, and important”. The group was formed through consulting staff who currently work in locked or inpatient settings at the hospital health service the supporting PhD research was conducted. Participants were invited to participate and those who were willing and available attended the session. Stakeholders were asked to read the practice principles and provided a brief overview of the research surrounding development. They were then invited to provide any feedback on likelihood of use, implementation, and general perspective of utility.

A thematic analysis was conducted using Braun and Clarke (2014) methods. Some of the key themes in the feedback included concerns with the shared responsibility of facilitating activity; support from a governance structure to implement activity and longevity of implementing the practice principles.

All of the nursing staff noted concern with the entire multidisciplinary team being responsible for providing leisure activity. Nurses report being overwhelmed with their current duties, raising concern they cannot do more with their time. All stakeholders suggested concern with support from their governance structure, in particular, funding resources or staff.

One staff member stated she did not like the inclusion of words such as ‘should’ or ‘must’. This suggestion was discussed with other stakeholders who believed ‘should’ is an important inclusion to provide urgency and a sense of need behind the principles.

Staff discussed concerns on the rollout and implementation of practice principles. Some suggested the need for people to ‘take ownership’. The psychiatrists identified the environment, positive risks, and consumer involvement in treatment goals as highly important.

### Practice principles

Ten principles have been formed more broadly so mental health inpatient units can interpret, adapt, and implement meaningful leisure activities for their consumers. The principles should cater to the individual needs of the consumers and aim to improve the consumer experience.

### Principle 1: Leisure is a health-creating and health-promoting activity that brings meaning and purpose to Life

The first principle sets the tone and establishes cause for the remainder of the principles. Leisure is salutogenic and health-creating has been established along with the many benefits of engagement (Caldwell, 2005; Lindström & Eriksson, 2005). The remainder of the principles suggests action and change that are required to create optimal care based on the evidence (Levick et al., 2022).

### Principle 2: A variety of Leisure activities should always be on offer and beyond business hours

Activity should always be on offer for consumers to have the freedom to engage in meaningful activity, particularly beyond business hours. Activities should closely represent the variety we would expect to see in the community (within reason) to promote volition, a sense of agency, and promote recovery. Participation in an activity of choice assists in developing healthy habits and routines in the community. Wykes et al. (2018) found consumers were more positive about their inpatient experience when presented with the opportunity to engage in meaningful activity, regardless of the severity of their illness.

### Principle 3: A positive amount of risk should be taken to allow Leisure participation

Risks can present as a perceived challenge and barrier to engagement. Risks can include perceived risk by staff and risk aversion from the organisation. Positive risk-taking suggests a small number of necessary risks improves the quality of life of consumers (Carr et al., 2004; Robertson & Collinson, 2011). Consumers can be enabled to engage in activities with small amounts of risk which promotes personal growth, autonomy, and opportunity for success (Robertson & Collinson, 2011). Risk is a part of everyday life and should be assessed, evaluated, and carefully considered as a normal part of operating a MHIU (Just et al., 2021). In the attempt to reduce risks, occupational opportunities are often minimised leading to occupational deprivation. Strategies to reduce risk should thoughtfully be considered and evaluated however, a level of risk can be tolerated without causing serious harm (Just et al., 2021).

Just et al. (2021) suggest organisations can support positive risk with training staff; supervision and reflective practice; a culture shift for new practice; review of policy and guidelines; review of workload demands and improve therapeutic relationships with consumers.

### Principle 4: Scheduled Leisure activities including individual and group programs should be offered every day

Consumers and staff both reported a lack of group activity offered which reduces the social opportunities (Levick et al., 2022). Lim et al. (2007) found there is limited group activity offered on MHIUs which can contribute to boredom and seldom activity. Furthermore, more than half of the consumers that participated in this study stated engagement in occupational therapy (individual or group programs) contributed to improved function in their daily life. There is varied evidence on the benefits of group-based activity in inpatient settings. However, Lloyd and Lee Williams (2010) findings suggest individual and group activity is a core domain for mental health occupational therapists. Evaluation and reflection of a group’s value is critical to providing effective treatment. Ultimately, consumers will gain a range of skills, social engagement, and meaningful engagement in any form of participation (Lloyd & Lee Williams, 2010).

The multidisciplinary team need to value the time, effort, and importance of engagement. To provide the opportunity for success in implementing a leisure program, a member or delegate from each discipline (psychiatry, nursing, occupational therapy, social work, and psychology) should champion and take lead to represent their discipline. A delegate from each discipline should be involved to plan the successful implementation of activity. A cultural shift needs to occur for this to be successful and support the quality of care for consumers (Lloyd & Lee Williams, 2010). Consumer engagement in therapies with all disciplines should be considered equally important. A suggestion is to schedule medical reviews and nursing-related tasks (such as blood work or observations) to provide consumers with the opportunity to engage without concern of missing out on leisure activities available. Clear appointment and routine times of engagement with the treating team may assist consumers to plan their day and reduce or frustration when waiting to be seen.

### Principle 5: Social engagement and meaningful conversation are invaluable for Leisure participation

Engagement in leisure activity is an important and adequate goal to have as part of treatment whilst being an inpatient. Staff should prioritise engagement with individual consumers as a necessary part of their role. Meaningful engagement and conversation were one of the main leisure activities identified by consumers (Levick et al., 2023). Leisure and social engagement should be prioritised as much as documentation, medication management, and other duties for all staff.

### Principle 6: The governance structure should reflect these Leisure-related principles as necessary and important evidence-based care

To make a change, the governance structure needs to reinforce the importance of leisure and recreation. The governance structure may include local, state, and national macro levels of health. Local governance structures can make meaningful and immediate changes to their staff’s role descriptions, strategic plans, incorporation of daily operations, and priorities to provide therapeutic modalities. Levick et al. (2022) suggested there is limited leisure-related language found in Australian and international legislation, which could be a barrier to engagement.

A balance between a top-down and bottom-up approach needs to occur to make a cultural shift (McDermott et al., 2015). A top-down approach would include the governance or directors of a mental health service filtering down changes to policy, procedures, and practice. This needs to occur to make meaningful change in mental health units and create influence over policymakers (McDermott et al., 2015). Directors and leaders need to identify leisure engagement as an important factor in consumers’ recovery to facilitate a culture change from the bottom level. The governance structure can assist with ongoing momentum for the multidisciplinary team to reduce the risk of failure or programs ceasing due to lack of interest. A bottom-up approach would include ground level staff or those with direct service provision of consumers. Staff would suggest necessary changes needed to assist staff with their role, specifically with increased resources, training opportunities, and staff ‘buy-in’ (McDermott et al., 2015).

### Principle 7: A monotonous and uninviting built environment prevents Leisure engagement and fosters boredom

The environment encompasses the physical environment (Taylor, 2017). Consumers have longitudinally reported an environment with no stimulation or meaningful activity is harmful to their mental state (Cutler et al., 2021). Typically, MHIUs are found to be not engaging with a limited occupational opportunity to occur naturally (Cutler et al., 2021). Suggestion for improvement on the built environment was highlighted by staff (Levick et al., 2022) and consumers (Levick et al., 2023) as a barrier to engagement. Liddicoat et al. (2020) stated there is a strong link between the built environment and wellbeing. Contemporary and good architectural design are known to provide better clinical outcomes, support recovery, and reduce stress for staff (Liddicoat et al., 2020). Key built environmental design elements should consist of access to natural light, artwork involving nature, outdoor areas or interior green spaces, sensory stimuli, ambient lighting (which has been shown to reduce anxiety), and a range of furniture to choose from (Liddicoat et al., 2020). The built environment should have adequate space to conduct group activities as well as an opportunity for self-directed individual activities.

Cutler et al. (2021) concluded consumers feel safer and there are reduced incidences of aggression when they have a sense of privacy (with locked bedroom doors) and good environmental design. Having the opportunity to engage in meaningful activity provides choice, promotes autonomy, and improves personal causation. Consumers report leisure activity improves the consumer experience and assists with staff satisfaction.

### Principle 8: Consumers should be involved in developing their Treatment goals and planning for Leisure activity

As part of recovery-oriented practice, consumers need to be involved in their treatment goals to support their recovery and wellbeing. Consumer involvement allows the expression of their ‘goals, wishes, and aspirations’ (Commonwealth of Australia, 2013). Consumer involvement in their goals is essential for successful treatment as it allows choice and personal causation which builds capacity to make informed choices of their own care. Of course, consumers involvement may differ at different stages of their care, for example at the beginning of involuntary treatment due to duty of care. Coffey et al. (2019) identified a strong link between perceived quality of care and recovery-oriented practice. Ultimately, the goal of recovery-oriented practice is to provide quality care and improve mental health outcomes and quality of life for consumers with mental health issues (Commonwealth of Australia, 2013).

### Principle 9: Documentation needs to reflect meaningful engagement and Leisure preferences to support Treatment

Recovery and participation should be measurable and specific as a record to support the development of therapeutic goals and considerations for discharge (Commonwealth of Australia, 2013). Recovery-oriented language should be exemplified through all areas of documentation including clinical notes, mental health act paperwork, and policy/procedures (Coffey et al., 2019; Commonwealth of Australia, 2013)). Implementing and utilising standardised tools and checklists as part of the admission process will support treating teams to make informed choices for consumer care.

Many treating teams currently utilise checklists and structured proforma documentation as part of the admissions process including belongings lists, general demographic information, risk assessment (including static and dynamic risk factors) (Desmarais et al., 2012), initial assessment (which may include the presenting problem, previous mental health history), physical health screens (such as metabolic screening tools), recovery goals and the list goes on. There is often limited discussion regarding the person’s current routine, habits, and interests that may assist in developing a rapport and understanding of the person-centred factors (Taylor, 2017). Not only are leisure interests helpful to assist in the creation and development of meaningful treatment goals; but can be used to guide therapy beyond an admission. At times there is limited meaningful information provided to a community health team regarding the contextual person factors that assist in treatment planning when discharged from an MHIU.

Tools and checklists can be used as a meaningful way to capture leisure interests. Tools such as the Checklist of Leisure Interests and Participation (CLIP) (Levick et al., 2022) may provide insight into activities for consumers. Individual inpatient units are encouraged to utilise tools or checklists such as the CLIP to explore occupational opportunities in their setting.

### Principle 10: Acute environments should have the necessary resources to provide genuine Leisure participation

Resources are inclusive of an adequate built environment, social environment (staff with appropriate level training), and equipment to engage in an activity.

Staff should be considered an important and critical resource (Brownie, 2011). Training should be offered to assist with group facilitation with consideration of specific therapies or skills required to host groups. Supervision and reflective practice should be considered an important tool for staff to consider improvement on facilitation of groups and fostering meaningful individual participation. The multidisciplinary team should consider their interests and skills as valuable therapeutic tools for facilitation.

A range of indoor activities should be always available for consumers. Consideration for funding to provide adequate resources and replacement if any are broken. Resources for daily activity should extend beyond board games and television (Cutler et al., 2021).

## Discussion

Leisure is an important therapeutic modality and the ongoing benefits in the inpatient unit and community. These recommendations suggest an increase in meaningful leisure activity may assist with the recovery of acutely unwell consumers. The principles aim to provide an evidence base and guidance on key areas required for meaningful leisure engagement. Macro-level changes may include improvements to local policy, service goals, risk assessment, and operational guidelines (Coffey et al., 2019). Meso-level changes may include staff training and changes to the built environment (Coffey et al., 2019). Micro-level changes may include further exploration of the consumer’s interests and evaluate methods to implement the activity in each MHIU (Coffey et al., 2019).

### Clinical application

There must be a cultural shift in the way mental health inpatient units are run to make meaningful change. This cultural shift needs to be adopted in a top-down approach starting from a director-level shift. This approach means directors need to be convinced that this is important for consumer recovery and then assist with the necessary resources or funding to make a change. For successful implementation, staff should be included for collaboration and discussion on potential challenges of implementing the leisure principles. This collaboration allows staff the opportunity to problem solve and express interest in development (Table 1).

**Table 1.** Key principles to support occupational engagement.

Principles
1. Leisure is a health-creating and health-promoting activity that brings meaning and purpose to life. *Engagement in activity assists with recovery. Leisure is an important therapeutic modality*
2. A variety of leisure activities should always be on offer and beyond business hours. *Activity should be as readily available as it would be in the community*
3. A positive amount of risk should be taken to leisure allow participation. *Activities should be freely available to consumers to provide opportunity to engage in meaningful leisure activity that they would typically have in their home environment*
4. Scheduled leisure activities, including individual and group programs, should be offered every day. *The responsibility of engagement should be shared amongst the entire multi-disciplinary team to provide optimal care. A champion from each discipline should support the facilitation of leisure*
5. Social engagement and meaningful conversation with consumers are invaluable for leisure participation. *This should be considered a necessary part of staff’s roles*
6. The governance structure should reflect these leisure-related principles as necessary and important evidence-based care. *Some of the areas this could be reflected include policy, strategic and operational plans, and role descriptions*
7. A monotonous and uninviting built environment inhibits leisure engagement, fosters boredom, and delays recovery. *The built environment should be inviting and ‘home-like’ to promote recovery*
8. Consumers should be involved in developing their treatment goals and planning for leisure activity. *A consideration of consumers interests, likes, and ambitions should be included to motivate and encourage participation*
9. Documentation needs to reflect meaningful engagement and leisure preferences to support treatment. *Leisure-related standardised tools and checklists should be used at intake and discharge as best practice*
10. Acute environments need to have the necessary resources to provide genuine leisure participation. *Resources should include physical materials and staff to support facilitation of leisure activity*

These principles should be considered a starting point for improving leisure in MHIUs. The original research the practice principles were based on focussed on MHIU’s and did not explore the application of these principles in outpatient or day rehabilitation settings. The practice principles are based on occupational therapy theory and recovery-oriented principles. The practice principles provide a basis for evidence-based therapy and likely to be translatable to a number of practice settings. Further research of implementation of leisure programs and evaluation of the principles included in this paper in clinical settings such as MHIUs, day rehabilitation and outpatient settings which would assist to establish their usability and efficacy for practice. Research should explore the impact the practice principles may have on consumer experiences, and the impact on instances of aggression, seclusion, and use of PRN medication.

### Future research

Health services should implement these practice principles to make an immediate change to conform to best-practice standards. Evaluation and implementation of the practice principles in clinical settings are required. The practice principles should be applied to an Australian adult mental health context for implementation and evaluation. Future research may include a case study of one or more inpatient units over a set period (e.g. two years) to explore contextual factors and ease of application with the practice principles. Some factors to be considered in future research may be an improvement of the application of leisure as a therapeutic modality (reviewing levels of engagement in proposed activity and an increase in meaningful occupation). It could be hypothesised that if there is an increase in leisure activity resulting in improved consumer engagement, there would be a reduced readmission rate and a reduction in serious aggression such as seclusion and restraint. The successful implementation of the practice principles will assist policymakers to determine the need for change to overarching legislation and policy that may be limiting meaningful engagement. Furthermore, these principles would assist policymakers and governance structures to determine the benefit of resources and funding necessary for improvements to units.

## Conclusion

Leisure activity is considered a valid, therapeutic, and meaningful activity to support the recovery of consumers with mental health issues. The consumers experiences can be improved through the implementation of leisure activity on MHIU. Ten leisure principles were developed based on the literature reviewed and the findings of research conducted throughout PhD research (Levick et al., 2022). The principles provide health services an opportunity to review the service they currently provide to consumers and determine whether they are providing a therapeutic service. The practice principles target the built environment, social environment, resources, and funding that is available, review of documentation styles, risk management, and the need for scheduled activities.

Leisure engagement is considered salutogenic and meaningful. Consumers should be provided the opportunity to engage in leisure activity as they would in their home environment. Cognitive stimulation and supportive staff are conducive to positive recovery opportunities.

The application of this theory suggested more occupational opportunities may enhance consumers’ recovery from mental illness on MHIU. Support to develop ground-level changes such as routine, access to a safe environment, and engagement in meaningful occupation can promote a therapeutic environment. The practice principles have the potential to be applied to MHIUs across Australia or in similar countries. Further research to explore the implementation and evaluation of these principles in Australia and other countries will assist with implementation guidelines for best practice. An inpatient unit could be used as a case study of implementation and a longitudinal review of the changes made within the unit related to seclusion and re-admission rate. Occupational therapists should harness and champion the rollout of the practice principles in their respective units to enhance occupational opportunities. For successful implementation of the practice principles, consumers, service providers (staff with direct service provision) and policymakers need to be involved in implementation and evaluation.

## Key points for occupational therapy

- Leisure can be a powerful therapeutic modality in MHIUs that facilitate recovery and engagement in meaningful occupation.
- Organisations need to provide therapeutic services and improve the quality of mental health inpatient services that currently exists to support recovery-oriented practice for people with severe and complex mental illness.
- Consumers should be included to review and change of MHIUs to provide care that supports their needs and interests. Implementation of the Levick’s Leisure Practice Principles will assist to reduce the rate of seclusion and violence within inpatient settings which in turn will provide better consumer outcomes.
